# High Frequency Electric-Field-Assisted Preparation of BN/Epoxy Resin Composites with Excellent Electrical, Thermal, and Mechanical Properties

**DOI:** 10.3390/polym17111429

**Published:** 2025-05-22

**Authors:** Dongyuan Du, Yanpeng Hao, Yunhua He

**Affiliations:** 1 School of Electric Power, South China University of Technology, Guangzhou 510640, China; 2 Power Science Research Institute of Yunnan Power Grid Co., Ltd., Kunming 650000, China; heyunhuank@hotmail.com

**Keywords:** electric field induction, BN orientation, epoxy resin, thermal conductivity

## Abstract

Since epoxy resin has excellent mechanical and insulating qualities, it is frequently utilized in dry transformers. Low thermal conductivity, however, has prevented it from be developed further in high-frequency and large-capacity dry transformers. This study describes the preparation of sheet boron nitride (BN)/epoxy resin composites with superior electrical, thermal, and mechanical properties through the application of varying strengths of high-frequency alternating-current (AC) electric fields. Using an optical microscope, the orienting process of BN in epoxy resin under an electric field was studied. In parallel, tests were conducted on the BN/epoxy resin composites made with varying electric field strengths. The findings indicate that the preparation using a high-frequency AC electric field decreases BN agglomeration and improves the composite’s properties. The overall performance is comparatively ideal when the applied electric field strength is 30 V/mm, the tensile strength is 48.3 MPa, the breakdown field strength is 37.65 kV/mm, and the thermal conductivity of the BN/epoxy resin composite is 0.95 W/(m·K). The thermal conductivity greatly increased and BN was organized into chains when the electric field approached 60 V/mm. The tensile strength and breakdown field strength, on the other hand, declined.

## 1. Introduction

The performance of insulation materials is especially crucial when power equipment advances toward higher voltages and frequencies and power grades improve [1]. Because of its excellent mechanical and insulating qualities, epoxy resin is frequently used in dry transformers, high-frequency transformers, and other power electronic equipment [2]. When the equipment is operating, the epoxy resin ages over time due to the combined effects of heat, electricity, and mechanical force, ultimately causing the power equipment to fail [3]. According to studies’ findings, epoxy resin materials with high thermal conductivity have a long service life and can prevent equipment overheating and premature aging [4,5,6].

The advantages of adding inorganic fillers to the epoxy resin matrix to improve its thermal conductivity include a low processing cost, an easy preparation process, and a convenient operation process [7,8,9]. Excellent thermal stability, strong chemical properties, high thermal conductivity, high resistance, and a low dielectric constant are all characteristics of boron nitride as an electrical insulator [10,11]. As a result, research on BN/epoxy resin composites, a novel kind of high-thermal-conductivity insulation composite, has accelerated.

Wang et al. [12] found that the thermal conductivity of the BN/epoxy composite could be increased to 1.52 W/(m·K), and that the mechanical properties first increased and then decreased with the content of BN. Jang et al. [13] found that the longer the silane coupling chain length, the more significant the improvement in the thermal conductivity of the composite. Huang et al. [14] studied the thermal conductivity and dielectric properties of epoxy filled with BN and spherical BN, and they found that BN improved the thermal conductivity of the epoxy more significantly, while spherical BN showed better mechanical and electrical properties.

The traditional method used in the above research needs a higher filling amount to improve the thermal conductivity of the composite material. The electric-field-induced particle self-assembly effect has been widely used to induce the orientation and chain arrangement of fillers in the matrix to enhance specific physical properties, providing a new way to explore the improvement of the thermal conductivity of composite materials [15,16,17].

Yang et al. [18] studied the orientation dispersion of montmorillonite in polyethylene induced by an electric field, along with its effect on its electrical dendritic properties. The results showed that montmorillonite (MMT) sheets deflect in a direction parallel to the electric field due to electric field induction, which also aids in the uniform dispersion of MMT sheets in low-density polyethylene (LDPE). Inhibiting electrical branches perpendicular to the directed electric field is a stronger suit for the induced LDPE/MMT composite. Belijar et al. [19] cured a liquid prepolymer composite that had been combined with epoxy resin and BaTiO_3_ by applying a consistent AC electric field. The findings demonstrate that creating particle chains can greatly increase the composites’ dielectric constant. Chao et al. [20] examined the extent to which various electric-field-induced orientations enhanced the material’s thermal conductivity. Carbon black and diamond sheets were oriented during the polymer curing process by applying DC and pulsed electric fields. Mi Yan et al. [21,22] studied the effects of pulsed electric fields of different intensity and frequency on the orientation degree of boron nitride nanosheets and the thermal conductivity of composites. They found that the orientation degree of BN tends to be saturated at higher electric field intensity, while the increase in the thermal conductivity of the composite does not lead to a saturation phenomenon. The literature [23,24] indicates that the thermal conductivity of the research materials plays a crucial role in the operation of the equipment. Among them, the size of the nanomaterials and the research methods provide references for this article.

BN/epoxy composites with different orientations and degrees of chain arrangement were prepared by applying high-frequency AC electric fields of different intensity. By observing the arrangement of BN in epoxy resin under electric fields and testing the thermal conductivity, tensile strength, dielectric constant, and breakdown field strength of BN/epoxy resin composites prepared with different electric field strengths, the effects of BN orientation degree on the thermal conductivity, mechanical properties, and electrical properties of BN/epoxy resin composites were analyzed.

## 2. Theoretical Basis

The induced dipole moment P of the dielectric ball in a uniform electric field is shown in Formula (1), and the torque of the induced dipole is shown in Formula (2). In addition, the orientation of the medium in epoxy resin needs to overcome the viscous resistance *F* and Brownian motion force *KT* [25].

(1) $$P={4\pi \varepsilon_{m}r}^{3}(\frac{\varepsilon_{p}-\varepsilon_{m}}{\varepsilon_{p}+2\varepsilon_{m}})E$$

(2) $$\vec{T}=\vec{P}\times \vec{E}$$

(3) $$F=6\pi r^{3}\eta \gamma$$

(4) $$\lambda =\frac{\pi \varepsilon_{0}\varepsilon_{m}r^{3}{\left(\beta E\right)}^{2}}{KT}$$

where *ε*_*p*_ is the dielectric constant of the medium, *ε*_m_ is the dielectric constant of the substrate material, *E* is the applied electric field strength, η is the viscosity coefficient of the substrate material, *γ* is the shear rate during the viscosity test, and *λ* is the ratio of induced torque to Brownian force.

**Figure 1:**
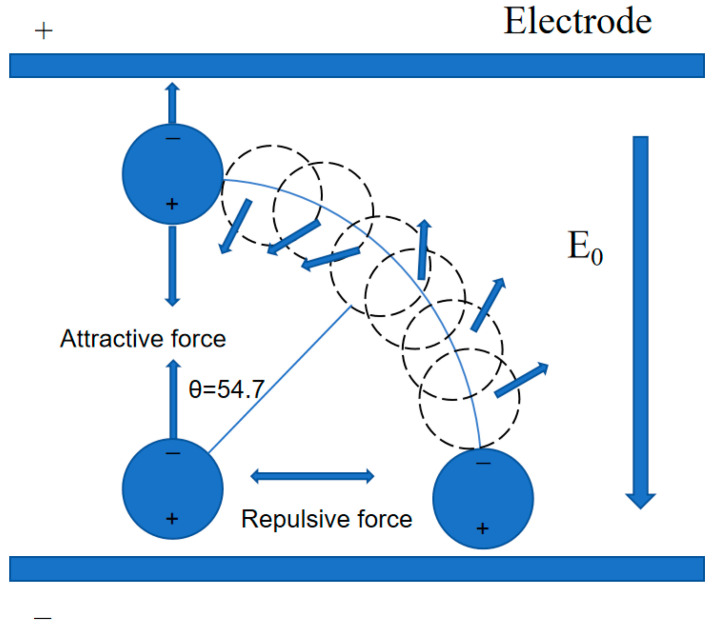
Schematic diagram of the interaction of electric dipoles under an electric field.

The analogous induction dipole of the particle itself will also create an uneven spatial electric field, which will cause a gradient force on the adjacent particle, in addition to the electrophoretic force produced by the electric field. The forces between the particles are computed using a two-particle system as an example (Figure 1). The induced dipole moment for spherical particles is parallel to the applied electric field. When an electric torque acts on non-spherical ellipsoid or cylindrical rod particles, the induced dipole moment in a stable equilibrium condition is parallel to the applied electric field. The spatial locations of two parallel, equal-sized electric dipoles are depicted in Figure 1, and the electric field produced by electric dipole 1 at position 2 is as follows [26,27,28]:

(5) $${\vec{E}}_{12}=\frac{1}{4\pi \varepsilon_{m}}(\frac{2P\cos\theta }{r^{3}}{\hat{r}}_{12}+\frac{p\sin\theta }{r^{3}}\hat{\theta })$$

The force on electric dipole 2 is as follows:

(6) $${\hat{F}}_{21}=\frac{3P^{2}}{4\pi \varepsilon_{m}r^{4}}((1-3\cos^{2}\theta ){\hat{r}}_{21}-\sin\theta \cos\theta \hat{\theta })$$

It is also observed that the dipoles attract each other when θ < 54.7° and repel each other when θ > 54.7°. The attraction aligns along the dipole with a magnitude of 3*P*^2^/(4πε_m_r^4^) when θ = 0; the repulsive force is aligned along the dipole with a magnitude of 3*P*^2^/(4πε_m_r^4^) when θ = 90°. This indicates that, under an electric field, the fillers in the liquid polymer will spontaneously assemble and finally link end-to-end in the direction of the electric field to form a chain arrangement.

## 3. Experiment

### 3.1. Experimental Material

**Figure 2:**
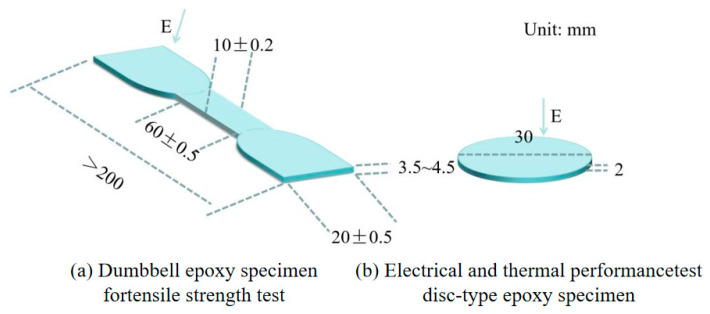
Schematic diagram of sample.

We used bisphenol A type E-51 epoxy resin (epoxy equivalent of 169–181 g/eq), methyl tetrahydrophthalic anhydride as a curing agent, micron sheet BN, and 2, 4, 6 tri (dimethylaminomethyl) phenol as an accelerator. The dimensions of the test samples for thermal conductivity and breakdown field strength are shown in Figure 2a, and the dimensions of the test samples for tensile strength are shown in Figure 2b.

### 3.2. Material Preparation

**Figure 3:**
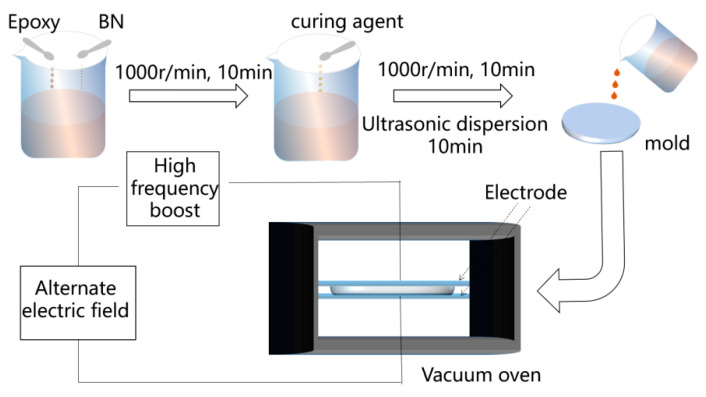
Flowchart of preparation of BN/epoxy resin composites by high-frequency electric fields.

The epoxy resin casting process is shown in Figure 3. To ensure that the BN and epoxy resin were equally mixed, BN and epoxy resin were first mixed (with BN making up 30% of the mixture) and swirled at 1000 revolutions per minute for 10 min. The equally mixed BN/epoxy resin composite suspension was then mixed with the curing agent (the curing agent/epoxy resin ratio was 1:1) and accelerator (the quantity was 2%) and dispersed for 10 min using an ultrasonic dispersion device. After 30 min of vacuuming, the resultant liquid was placed into the mold. The two electrodes were heated to 100 °C in 30 min, cured for 10 h, and then heated to 120 °C for an additional 10 h. A high-frequency AC electric field of 10 kHz was applied to the electrodes and maintained for 30 min. The samples were labeled as follows: 0&BN-EP without an applied electric field, 15&BN-EP with 15 V/mm, 30&BN-EP with 30 V/mm, and 60&BN-EP with 60 V/mm. This was based on the application of high-frequency electric fields of varying intensities. Polytetrafluoroethylene material was used around the mold, and an electric field was applied to the steel plate on both sides.

### 3.3. Performance Test

The dispersion and orientation of BN in epoxy resin under different electric field intensities were observed by a Sirion 200 scanning electron microscope.

Tensile specimens were prepared according to the standards [22] GB/T 2567-2021 and ASTM E132-17 to test the tensile strength of the epoxy resin composites. Tensile testing was carried out using a CMT5105 microcomputer-controlled electronic universal testing machine for SANS. In order to eliminate the test error, the test loading speed was 0~50 N 2 mm/min and 50~800 N 0.5 mm/min.

The epoxy sample for dielectric property testing was prepared according to the standard GB/T 31838.6-2021, at a size of Φ30 × 2 mm. The relative dielectric constant and dielectric loss factor at room temperature were measured by a wide-band dielectric spectrometer (instrument model: ALPHa-ANB) with a column–column electrode (diameter: Φ20 mm, temperature −150 °C~350 °C, frequency 10^−6^~10^9^ Hz, impedance range 0.01 Ohm~100 TOhm, phase-difference accuracy 2 × 10^−3^, loss accuracy 3 × 10^−5^).

According to the standard [22] GB/T 1408.1-2016, the power frequency breakdown voltage of the sample was tested using an MLTC series AC voltage complete set under laboratory conditions.

## 4. Experimental Results and Analysis

### 4.1. Microscopic Morphology

**Figure 4:**
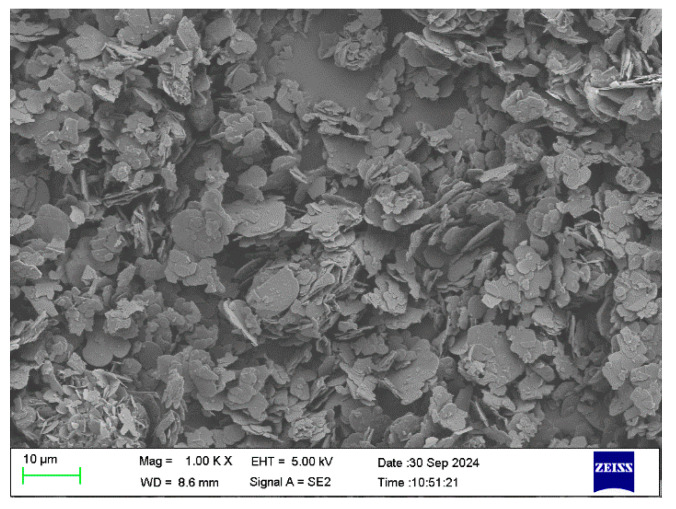
SEM scan of BN.

**Figure 5:**
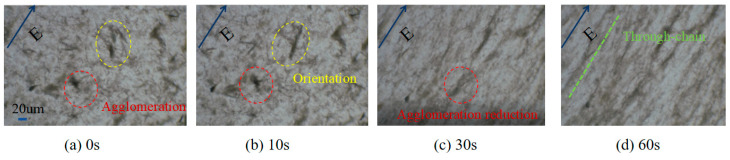
Optical microscope image of BN arrangement evolution over time under a 60 V/mm electric field.

**Figure 6:**
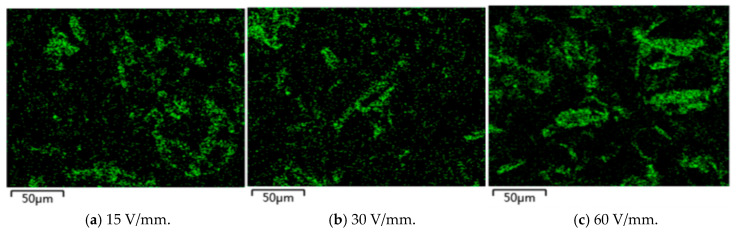
Analysis of EDS of boron nitride under different electric fields.

To determine the size of the BN, SEM scanning was adopted, as shown in Figure 4 specifically. Figure 5 shows the arrangement trend of BN over time under a 60 V/mm electric field. When no electric field was applied, the BN scattered and dispersed in the epoxy resin, and some BN appeared to agglomerate. After the electric field was applied, the BN began to orient under the action of the electric field force. When the electric field was applied for 10 S, some BN particles could be seen to turn. Upon applying an electric field for 30 s, the BN essentially finished orienting, and the combined BN was dispersed. The orientation degree of the BN was improved and a structure resembling a chain was formed when the electric field was applied for 60 S. The BN chain thickened when the electric field was applied more intensely. The liquid polymer’s particles rotated around their center of mass due to the torque generated by the electric field. The particles also experienced viscous resistance from the matrix at the same time. The balance between the two determines the rotational angular velocity. The torque of the BN rises as the strength of the electric field increases, and this leads to deflection. During the deflection process, the angle between the BN and the direction of the electric field strength decreases, and the torque decreases along the component perpendicular to the surface of the BN, and then gradually tends to be parallel to the direction of the electric field. The BN showed homogeneous and isolated dispersion in epoxy resin. With the increase in the electric field intensity, the orientation degree of the BN in the composite material increased. To determine the arrangement of the BN after pouring, EDS scanning was adopted, as shown in Figure 6. The greater the applied electric field, the better the chain-like arrangement of the BN.

**Figure 7:**
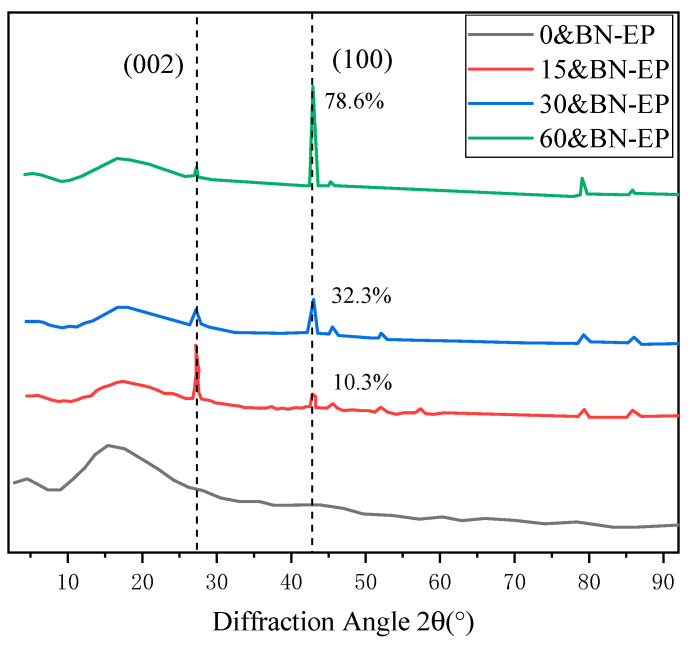
X-ray diffraction analysis of boron nitride/epoxy resin samples prepared under different applied electric fields.

When no electric field was applied, the strength ratio of the randomly distributed BN/EP composite material prepared was almost negligible. Under the action of a 15 V/mm electric field, the strength ratio of the oriented composite material increased to 10.3%, but at this time, the (002) peak still dominated, indicating that the orientation degree of the BN was relatively small under this electric field. As shown in Figure 7, with the increase in the electric field, the intensity ratio of the diffraction peaks of the composite material gradually increased, and at 60 V/mm, the intensity ratio increased to 78.6%.

### 4.2. Thermal Conductivity

**Figure 8:**
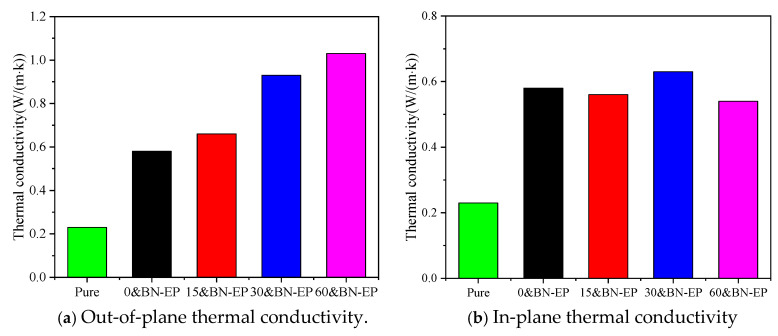
Thermal conductivity of BN/epoxy resin with different electric field strengths.

**Figure 9:**
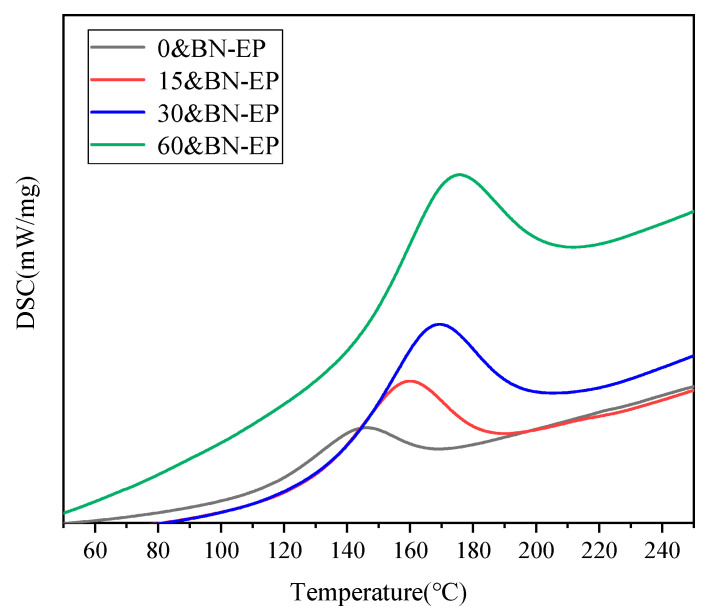
DSC of BN/epoxy resin with different electric field strengths.

The results of tests on the thermal conductivity of composite and pure epoxy materials are displayed in Figure 8, illustrating how the composite’s thermal conductivity rises with the applied electric field strength. Pure epoxy resin has a thermal conductivity of only 0.23 W/(m·K), whereas 0&BN-EP has a thermal conductivity of 0.58 W/(m·K). At a 60 V/mm electric field intensity, 60&BN-EP reaches 1.03 W/(m·K) thermal conductivity. Its thermal conductivity is 1.77 times higher than that of 0&BN-EP and 4.47 times higher than that of pure epoxy resin. This suggests that adding the induced electric field during the preparation process can significantly increase the thermal conductivity of epoxy composites. The composite’s out-of-plane thermal conductivity is greatly increased with the addition of BN; the thermal conductivity of 0&BN-EP is 0.58 W/(m·K), 2.52 times that of epoxy resin. This is mostly due to the BN lattice’s ability to prolong phonon free transit, lessen phonon scattering, and improve thermal conductivity. Following the application of a certain electric field, BN progressively aligns itself in the direction of the electric field. It is possible that certain sections of the BN are interconnected, creating a thermal conduction pathway that allows heat to be transferred. From the analysis of in-plane thermal conductivity, it can be seen that the electric-field-driven self-assembly of BN has little effect on the in-plane thermal conductivity of epoxy resin. This is mainly because the increase in the thermal conductivity of BN itself is not related to the strength of the electric field applied. Meanwhile, we conducted the glass transition temperature tests on the epoxy resin samples prepared under different electric fields, as shown in Figure 9. The glass transition temperature of the epoxy resin samples without an applied electric field was 144.2 °C. After applying an electric field, the glass transition temperature increased. With an electric field of 15 V/mm, 30 V/mm, and 60 V/mm, the glass transition temperatures of the epoxy resins were 159.6 °C, 168.8 °C, and 175.2 °C respectively. This is because the electric field drives the chain-like arrangement of BN, forming a conductive pathway, and thus increasing the glass transition temperature of the material.

### 4.3. Mechanical Properties

The universal tensile machine tested the critical load at break of the sample, and the tensile strength calculation is shown in Formula (7):

(7) $$\sigma =\frac{F}{S}$$

**Figure 10:**
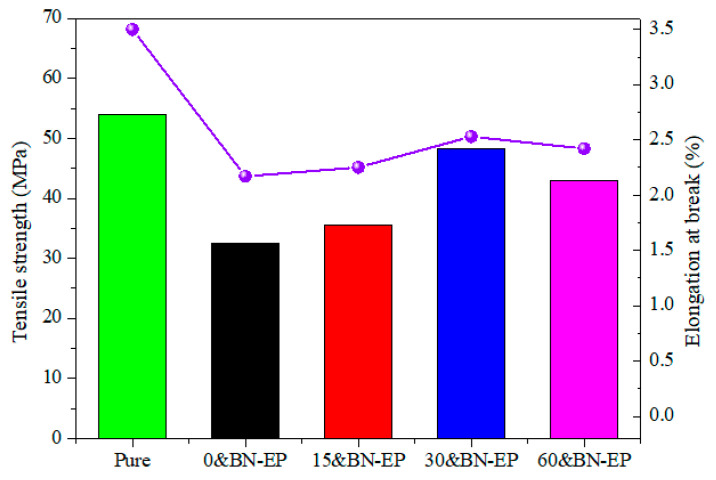
Tensile strength of BN/epoxy resin with different electric field strengths.

As can be seen from Figure 10, the tensile strength σ of the samples 0&BN-EP, 15&BN-EP, 30&BN-EP, and 60&BN-EP was 32.4 MPa, 35.6 MPa, 48.3 MPa, and 42.9 MPa, respectively. After the application of the electric field, the elongation at break of the epoxy resin in all samples decreased, and as the applied electric field increased, the elongation at break changed little. This is mainly because the brittleness of the material is related to the concentration of BN and has little to do with the applied electric field. With the increase in the electric field strength, the tensile strength of the composite first increases and then decreases. This is because the BN sample without an electric field was not evenly distributed in the epoxy resin, and partial agglomeration occurred. The agglomeration of BN increases the stress defects of the sample. When an electric field is applied, the induced dipole of BN also generates an uneven spatial electric field, creating a gradient force on nearby particles. The interaction between the dipoles reduces the reunion of the BN. The greater the electric field intensity, the greater the force generated between the dipoles, and the more obvious the BN dispersion. As the applied strength of the electric field continues to increase, BN essentially completes the orientation and gradually forms a long chain, resulting in a continuous interface between the BN and epoxy resin, and the tensile strength begins to decline perpendicular to the electric field’s direction.

### 4.4. Electrical Properties

#### 4.4.1. Dielectric Constant

**Figure 11:**
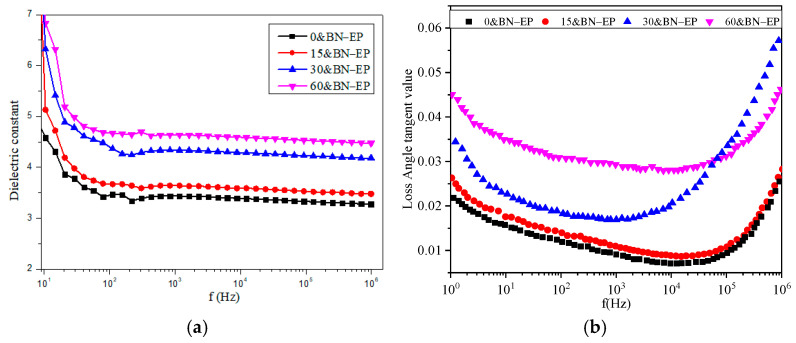
Dielectric spectrum of boron nitride/epoxy resin composites: (**a**) Real part of dielectric constant. (**b**) Dielectric loss tangent value.

The variation curve of the epoxy composite material’s dielectric constant (εr) and the dielectric loss tangent angle with frequency is displayed in Figure 11. The dielectric constant of epoxy resin composites rises as the strength of the electric field increases. On the one hand, adding BN to epoxy resin will result in a significant increase in the number of organic–inorganic interfaces. Additionally, there is a strong interaction between the functional groups of the two materials in the interface region, which intensifies the interface polarization and raises the composite material’s dielectric constant. On the other hand, if the packing particles and the substrate are regarded as microcapacitors, the packing particles change from random distribution to a chain arrangement along the electric field, which means that the microcapacitors change from the original series to parallel, the equivalent capacitance at both ends of the circuit will increase, and the increase depends on the difference in dielectric constant between the packing and the substrate.

Because the dielectric relaxation frequency of interfacial polarization is low, the interfacial polarization decreases and the degree of polarization weakens when the frequency increases. Therefore, the dielectric constants of composite materials all decrease with the increase in frequency and tend toward a fixed value. The sample preparation assisted by electric fields resulted in an increase in dielectric loss. This might be because the electric field treatment increased the number of BN/epoxy resin interfaces, and the corresponding interface polarization led to an increase in the loss of the composite material.

#### 4.4.2. Breakdown Field Strength

**Figure 12:**
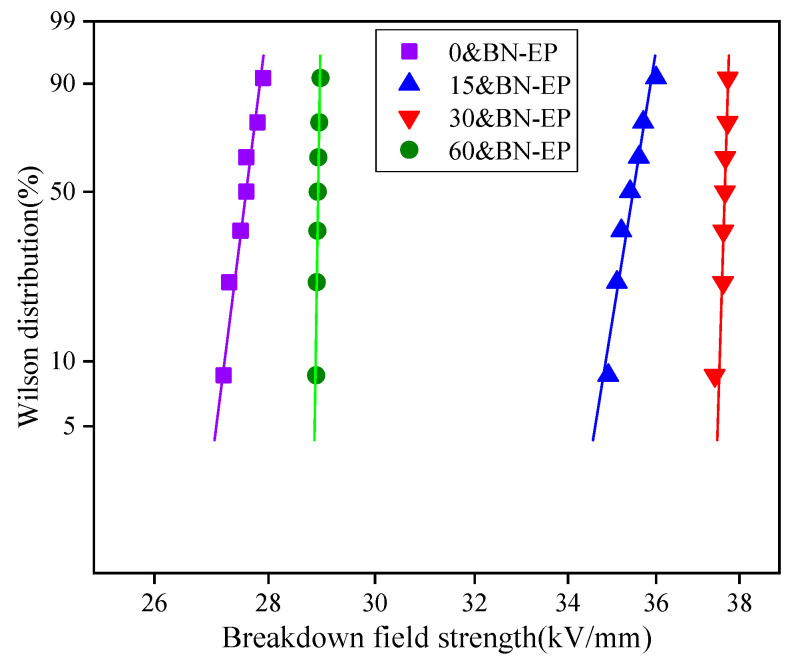
Breakdown field strength of BN/epoxy resin with different electric field strengths.

**Figure 13:**
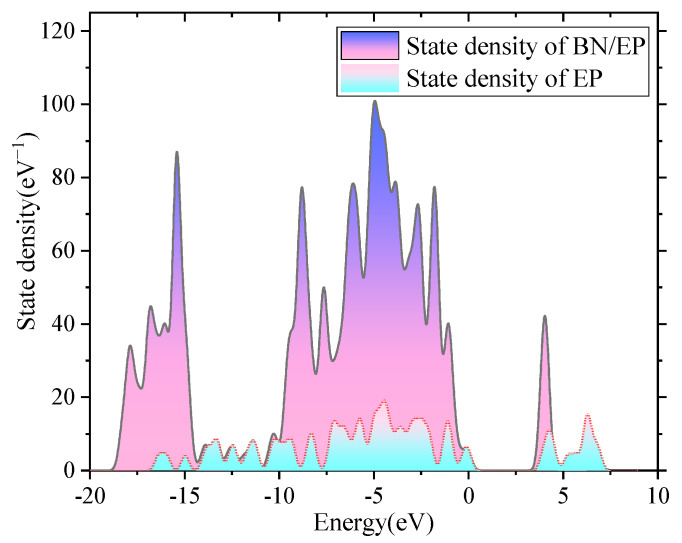
Epoxy resin and BN/epoxy resin state density.

Figure 12 shows the breakdown field strength of boron nitride/epoxy resin under different electric field intensities. With the increase in the applied electric field, the breakdown field strength of the BN/epoxy resin composites first increases and then decreases. The average breakdown field strength of the 0&BN-EP, 15&BN-EP, 30&BN-EP, and 60&BN-EP samples was 27.63 kV/mm, 35.43 kV/mm, 37.65 kV/mm, and 28.94 kV/mm, respectively. BN has a strong scattering effect on high-energy carriers accelerated by an electric field, which increases the breakdown field strength of the composites. Figure 13 shows the state density of epoxy resin and boron nitride/epoxy resin. By simulating the state density of BN and epoxy resin, it was found that the addition of BN introduces a new electron trap. The depth of the electron trap hinders the movement of free electrons in epoxy resin, so that the breakdown performance of the epoxy resin is improved.

The orientation of the BN after orientation is consistent, which reduces the scattering effect of high-energy carriers in all directions, and the breakdown field strength is slightly reduced. When the applied electric field intensity reaches a certain strength, the mechanical and electrical properties of the composite materials decline.

## 5. Discussion

**Figure 14:**
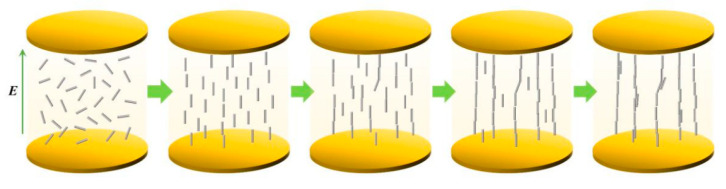
Schematic diagram of self-assembly of BN in an alternating electric field.

As can be seen from Figure 14, BN forms a conductive path under the action of an electric field, thereby enhancing the thermal conductivity of epoxy resin. However, the uniformity of the BN decreases, leading to the decline in its mechanical and electrical properties. Therefore, it is particularly important to explore the optimal field strength that can enhance the thermal conductivity of epoxy resin while maintaining good electrical and mechanical properties.

**Figure 15:**
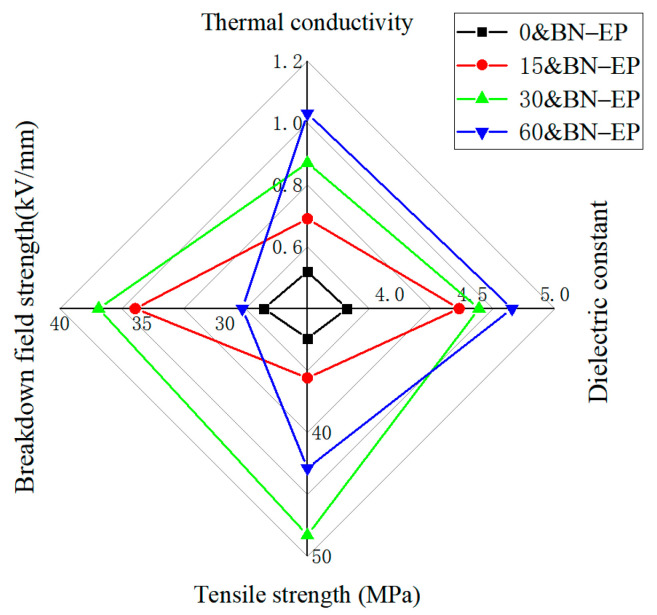
Radar diagram of thermal conductivity, tensile strength, dielectric constant, and breakdown field strength of BN/epoxy resin with different electric field strengths.

The comprehensive electrical, thermal, and mechanical properties of BN/epoxy resin composites were analyzed, and the radar diagram (Figure 15) of the thermal conductivity, tensile strength, dielectric constant, and breakdown field strength of composites prepared by different electric fields was drawn. It can be seen from the radar chart that the thermal conductivity increases with the increase in the applied electric field, and the tensile strength and breakdown field first increase and then decrease with the increase in the applied electric field. When the applied electric field strength is 30 V/mm, the thermal conductivity of the BN/epoxy resin composite is 0.95 W/(m·K), the tensile strength is 48.3 MPa, the breakdown field strength is 37.65 kV/mm, and the comprehensive performance is relatively optimal.

The BN/epoxy composite material prepared by high-frequency electric fields meets the requirements of 18~22 kV/mm breakdown field strength for epoxy resin used in dry transformers, and the thermal conductivity is improved, providing a reference for solving the problem of uneven temperature rise in large-capacity dry transformers.

## 6. Conclusions

In this study, the preparation method of high-performance BN/epoxy resin composite was studied, the orientation of BN in epoxy resin was observed by optical microscopy, and the thermal conductivity, mechanical, and electrical properties of BN/epoxy resin composites were tested experimentally. The results show the following:

(1) This work proposes a high-frequency AC electric-field-assisted preparation technique for high-performance BN/epoxy resin composites. The BN will experience torque from the induced dipole under the 10 kHz AC electric field, which will eventually cause the BN to orient along the direction of the electric field during the BN/epoxy resin curing process. By using this technology, the tensile strength is 48.3 MPa, the breakdown field strength is 37.65 kV/mm, the applied electric field strength is 30 V/mm, the thermal conductivity of the BN/epoxy resin composite is 0.95 W/(m·K), and the complete performance is comparatively ideal.

(2) The mechanism of high-performance preparation of BN/epoxy resin composites assisted by a high-frequency AC electric field is described. The BN agglomeration is reduced during the preparation assisted by a high-frequency AC electric field. The optical microscopy results of the BN’s arrangement evolution over time under electric fields show that there is local BN agglomeration when no electric field is applied, and the BN agglomeration decreases with the electric field over time. After the electric field is applied, the equivalent induction dipole of the BN itself will also produce an uneven spatial electric field, which will produce mutual repulsion on the nearby BN, reducing the agglomeration.

(3) This paper elucidates the mechanism of high thermal conductivity of BN/epoxy resin composites assisted by high-frequency AC electric fields. The lattice of BN makes the free travel of phonon movement longer, reduces the scattering effect of phonons, and enhances the thermal conductivity. After the electric field, BN gradually tends to align in the direction of the electric field under the action of the electric field. As the electric field’s strength increases, parts of the BN are connected to each other to form a thermal conductivity path, and heat can be conducted through the thermal conductivity path at this time, significantly improving the thermal conductivity.

(4) This paper elucidates the mechanism by which the mechanical and electrical properties of BN/epoxy resin composites are first increased and then decreased with the assistance of high-frequency AC electric fields. After the electric field is applied, the BN agglomeration phenomenon is reduced, the stress defects are reduced, and the mechanical properties of the composite are improved. As the electric field increases, BN forms a long chain along the direction of the electric field. There are interface defects between the BN and epoxy resin, and the mechanical properties decline. BN has a strong scattering effect on high-energy carriers accelerated by electric fields, which increases the breakdown field strength of the composites. The orientation of BN after orientation is consistent, which reduces the scattering effect of high-energy carriers in all directions, and the breakdown field strength is slightly reduced. When the applied electric field intensity reaches a certain strength, the mechanical and electrical properties of the composite materials decline.

## Data Availability

The original contributions presented in this study are included in the article. Further inquiries can be directed to the corresponding author(s).
